# Impact of *Sclerotinia sclerotiorum* Infection on Lettuce (*Lactuca sativa* L.) Survival and Phenolics Content—A Case Study in a Horticulture Farm in Poland

**DOI:** 10.3390/pathogens12121416

**Published:** 2023-12-02

**Authors:** Violetta Katarzyna Macioszek, Paulina Marciniak, Andrzej Kiejstut Kononowicz

**Affiliations:** 1 Laboratory of Plant Physiology, Department of Biology and Plant Ecology, Faculty of Biology, University of Bialystok, 15-245 Bialystok, Poland; 2 Wiesław and Izabela Królikiewicz Horticulture Market Farm, 97-306 Majków Średni, Poland; 3 Faculty of Biology and Environmental Protection, University of Lodz, 90-237 Lodz, Poland; 4 Department of Plant Ecophysiology, Faculty of Biology and Environmental Protection, University of Lodz, 90-237 Lodz, Poland; andrzej.kononowicz@biol.uni.lodz.pl

**Keywords:** anthocyanins, epidemiology, flavonoids, vegetation season, lettuce, phenolics, soil-borne fungus, temperature

## Abstract

*Sclerotinia sclerotiorum* is a cause of a prevalent and destructive disease that attacks many horticultural food crops, such as lettuce. This soil-borne necrotrophic fungal pathogen causes significant economic losses in worldwide lettuce production annually. Furthermore, current methods utilized for management and combatting the disease, such as biocontrol, are insufficient. In this study, three cultivars of lettuce (one Crispy and two Leafy cultivars of red and green lettuce) were grown in central Poland (Lodz Voivodeship), a widely known Polish horticultural region. In the summer and early autumn, lettuce cultivars were grown in control and *S. sclerotiorum*-infected fields. The lettuce cultivars (*Templin*, *Lollo Rossa*, and *Lollo Bionda*) differed phenotypically and in terms of the survival of the fungal infection. The Crispy iceberg *Templin* was the most susceptible to *S. sclerotiorum* infection compared to the other cultivars during both vegetation seasons. The total content of phenolic compounds, flavonoids, and anthocyanins varied among cultivars and fluctuated during infection. Moreover, phenolic content was affected by vegetation season with alterable environmental factors such as air temperature, humidity, soil temperature, and pH. The most increased levels of phenolics, both flavonoids and anthocyanins in infected plants, were observed in the Leafy red *Lollo Rossa* cultivar in both crops. However, the highest survival/resistance to the fungus was noticed for *Lollo Rossa* in the summer crop and *Lollo Bionda* in the autumn crop.

## 1. Introduction

Lettuce (*Lactuca sativa* L.) belongs to the largest family of dicotyledons, i.e., the *Asteraceae* family, and it is an annual self-pollinating plant [1,2]. Lettuce comprises a straight stem, the height of which ranges from 30 to 100 cm. Leaves are arranged in a spiral pattern and may form a dense rosette or head. They can be oblong, round, triangular, or transversely elliptical. Lettuce forms a deep root with horizontal lateral roots for maximum water and nutrient uptake [1]. There are six main types of lettuce: Crispy, Butterhead, Romain, Leafy, Stem, and Latin. Moreover, each type has a great variety, including its color and shape [3].

Lettuce is a leafy vegetable that is widely consumed across the globe, particularly in Europe, Asia, and North and Central America [1,4], due to its traditional and healthy qualities [5]. In salads or sandwiches, lettuce is typically consumed fresh and raw [6], as whole heads or cut leaves of lettuce are available on the market [7]. Fresh lettuce is rich in bioactive compounds such as phenolics and fiber [8,9,10], carotenoids, and tocopherol [9], as well as vitamins C, B1, B2, PP, and K [11], and minerals such as iron, calcium, and magnesium [10]. Lettuce leaf tissues contain phenolic compounds responsible for their health-protecting and antioxidant properties. Two main phenolic compound groups in lettuce are caffeic acid derivatives and flavonols (flavonoid class). Red lettuce cultivars contain high levels of another flavonoid class, anthocyanins [12,13]. Phenolic content depends on a variety and even cultivars during normal development under physiological conditions. Many studies have shown that the concentration of phenolics alters in plant tissues due to variable environmental factors such as light intensity or color [14]. Furthermore, the reduction in phenolic content in some cultivars is affected by processing and storage [15]. It is thought that lettuce polyphenols may have a more substantial antioxidant effect than vitamins C and E [16].

Despite the relatively short growing season, lettuce can be grown outdoors in the field and caterpillar tunnels and indoors in a greenhouse hydroponically [7]. Therefore, this vegetable is available to consumers all year. Lettuce is cultivated outdoors in temperate climate zones from early spring to early autumn. The optimal temperature range for lettuce growth is between 12 and 24 °C, as it is a cool-season crop [17]. Cultivating lettuces in the field employs various soil classes, with the optimal soil pH value ranging from 6.0 to 6.8 [18]. However, growing outdoors or indoors, lettuce crops are exposed to many pathogens, especially pathogenic fungi such as downy mildew (*Bremia lactucae*) [19], powdery mildew (*Erysiphe cichoracearum*), gray mold (*Botrytis cinerea*) [20,21,22], and white mold (*Sclerotinia sclerotiorum*) [22,23,24]. Among fungus-induced lettuce diseases, *S. sclerotiorum* is considered the most destructive one, and it causes stem rot, often called lettuce drop or white mold. *S. sclerotiorum* belongs to the division *Ascomycota*, family *Sclerotiniaceae*, and this necrotrophic fungus is a soil-borne polyphage that infects almost 600 plant species worldwide [25]. Its most important and preferred hosts are dicotyledons such as sunflower, oilseed rape, soybean, bean, pea, lentil, chickpea [24], potato, lettuce, carrot, cabbage, celery, pepper, poppy seeds [26], and from monocotyledons onions and tulips are at risk [25]. Dicotyledonous plants, such as lettuce, are more susceptible to the fungus because it secretes pathogenicity determinants such as oxalic acid (OA) and necrosis- and ethylene-inducing peptide 1-like proteins (NLPs) [27]. Oxalic acid suppresses host defense responses because it does not metabolize in dicotyledons but detoxifies in monocotyledons [27,28,29]. NLPs elicit cell death by binding to specific membrane lipids-glycosyl inositol phosphoryl ceramides (GIPCs) characteristic for dicotyledons but absent in monocotyledons [27]. One of the factors determining the fitness and difficulties in the management of *S. sclerotiorum* infection of lettuce is that up to 90% of the life cycle of this fungus takes place in the soil in the form of sclerotia [28]. Sclerotia serve as survival structures and can survive in the soil for up to eight years. Depending on the host plant, sclerotia can vary in size and shape [30]. However, contrary to *S. minor*, the disease caused by *S. sclerotiorum* is not correlated with the number of sclerotia in the soil [23]. The fungus can spread through contact from an infected plant to another healthy one [22,24]. In lettuce, *S. sclerotiorum* infection becomes visible when the rotting and decaying of the tissues begins at the stem, near the soil. Emerging lesions then spread down the plant until the roots break down. The watery spots also develop on the leaves and spread to the stem [23]. Sclerotia appear on the leaf surface close to mycelium and damage host tissues [22].

Complete lettuce resistance to *S. sclerotiorum* is lacking and to this date no genetic source of resistance is known [31,32]. The lower level of lettuce susceptibility to the fungus is rather based on developmental traits, such as, among others, stem mechanical strength, low leaf area or rapid bolting [31], and variable fungal factors such as ascospore and mycelium density [33]. Physiological traits, including phenolic compounds content, are rarely investigated in *S. sclerotiorum*-infected lettuce [34].

The aim of this study, performed under field conditions in a market farm in central Poland, was to estimate the influence of *S. sclerotiorum* on three lettuce cultivars during two vegetation seasons (summer and early autumn) in 2016. The impact of the *S. sclerotiorum*-induced disease on the lettuce cultivars’ survival and changes in their phenolic content, such as total phenolic compounds, flavonoids, and anthocyanins, were investigated. To our knowledge, this is the first study to show spontaneous *S. sclerotiorum* infection without additional artificial plant inoculation in functioning farm-producing food crops.

## 2. Materials and Methods

### 2.1. Lettuce Cultivars, Planting, and Cultivation

In all experiments, two varieties of lettuce (*Lactuca sativa* L.) were used Crispy and Leafy one Crispy iceberg lettuce *Templin* and two cultivars of Leafy lettuce: green *Lollo Bionda* and red *Lollo Rossa*. Iceberg lettuce *Templin* is fragile but has a perfect head shape, and it can be harvested easily from spring to autumn due to its raised and compact base. Leafy lettuces *Lollo Bionda* and *Lollo Rossa* are recommended for cultivation in the ground throughout a whole growing season, and they can be sold as heads or as ready-to-eat leaf mixtures.

**Figure 1:**
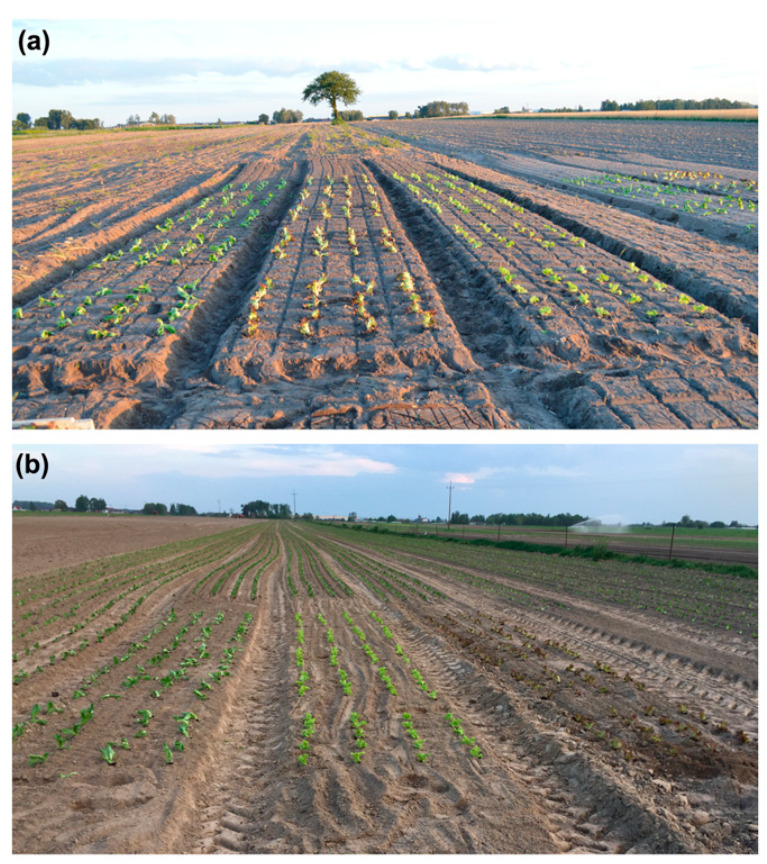
Establishment of the crops in the 2016 vegetation seasons. Seven-week-old seedlings: (**a**) the summer (1st) crop, from the left *Templin*, *Lollo Rossa*, and *Lollo Bionda* in the infected field; (**b**) the autumn (2nd) crop, from the left *Templin*, *Lollo Bionda*, and *Lollo Rossa* in the control field.

The lettuces were grown in the Wiesław and Izabela Królikiewicz Horticulture Market Farm in Majków Średni, in central Poland (Grabica Commune, Piotrków Trybunalski District, Łódź Province; 51°25′57.5″ N, 19°36′04.5″ E). Commercially available lettuce cultivated for production as human food has to be purchased from certified companies and grown according to agricultural rules and standardized practices. Therefore, lettuce seeds of *Templin* have been purchased from the Rijk Zwaan (Blonie, Poland), and *Lollo Bionda* and *Lollo Rossa* from the Nunhems Company (Warsaw, Poland); germination and production of young seedlings have been performed by the Schwanteland Jungpflanzen GmbH (Oberkrämer, Germany). Six-week-old seedlings have been planted in two crops - in summer and early autumn, from July 2016 until October 2016. In each crop, plants were grown in two experimental fields, one infected field containing *S. sclerotiorum*-contaminated soil and another, a control field located in the uninfected field in close proximity to the infected field. The individual lettuce cultivars were grown in beds of 8 m long and 1 m wide. Additionally, each bed was divided into 5 sectors, and in each sector, 20 lettuce seedlings were planted in 4 plots, with 5 seedlings per plot. Therefore, in each crop, 100 plants per bed of every cultivar were grown in the control field and 100 plants in the infected field (Figure 1). The beds were formed one day after the application of fertilizers. For each field (control and infected), 5 kg of ammonium nitrate, 5 kg of potassium sulfate, and 5 kg of triple super phosphate were applied. No additional applications of fertilizers were used during any of the vegetation seasons.

**Figure A1:**
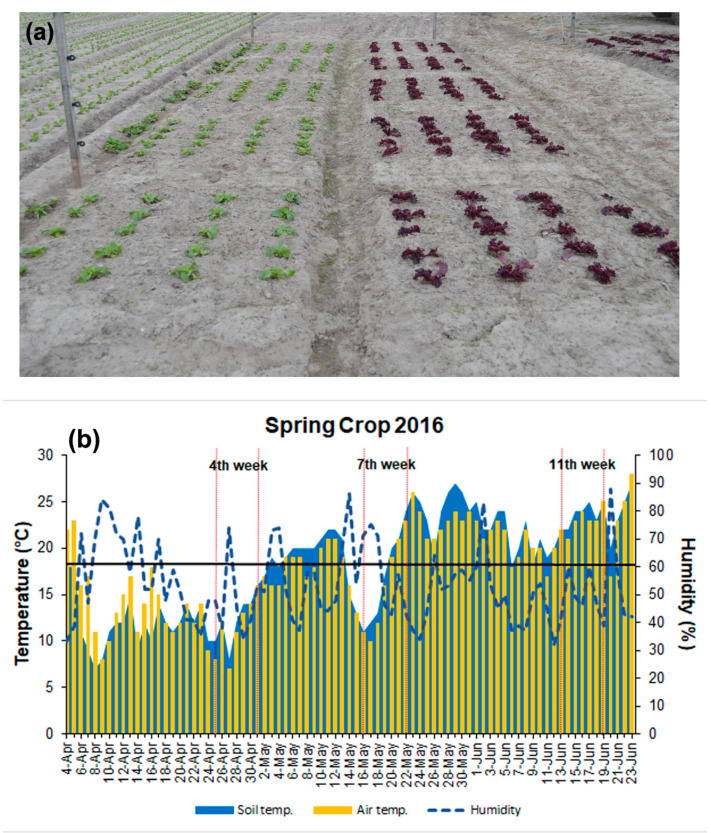
Establishment of the spring crops in 2016. (**a**) Seven-week-old seedlings in the infected field, from the left Crispy *Diamentinas* and Leafy *Lollo Rossa*; (**b**) soil and air temperature, and relative air humidity in the spring crop.

All experiments performed on the market farm started in the spring of 2016. However, the spring vegetation season lasted 81 days due to unfavorable weather conditions. Moreover, this crop was established with two cultivars-iceberg *Diamentinas* and Leafy *Lollo Rossa*. Thus, it could not be compared with two other seasons in 2016. Therefore, the spring vegetation season was not considered in this study (Appendix A, Figure A1).

#### 2.1.1. The Summer (1st) Crop, 2016

The summer crop was established by planting three cultivars of lettuce in the infected field: Crispy lettuce *Templin*, Leafy green lettuce *Lollo Bionda*, and Leafy red lettuce *Lollo Rossa* (Figure 1a). Control plants were planted in the uninfected field right next to the *S. sclerotiorum*-contaminated field. Plant irrigation with a net of sprinklers was carried out as a standard practice throughout the entire cultivation period, i.e., from 7 July to 18 August 2016 (for 43 days).

#### 2.1.2. The Autumn (2nd) Crop, 2016

This crop was established with the same parameters and lettuce cultivars as the summer one (Figure 1b), and it was carried out from 3 September to 22 October 2016 (for 50 days).

### 2.2. Air and Soil Parameters

From the first day of cultivation during both vegetation seasons, daily measurements of soil temperature and pH were carried out in the control and infected plots at 3 p.m. CEST, using a Flo 4in1 Soil Survey Instrument (Toya, Wroclaw, Poland). However, data on air temperature and humidity have been collected from the Polish website interia.pl, which publishes meteorological parameters obtained from the meteorological stations for every specific area/place in Poland every hour of the day (e.g., https://pogoda.interia.pl/prognoza-szczegolowa-majkow-sredni,cId,20064 (accessed on 30 October 2023)).

### 2.3. S. sclerotiorum Soil Contamination and Re-Isolation of the Fungus

The lettuce plants infected with *S. sclerotiorum* on the farm were first observed in early autumn of 2015 in a small part of the lettuce field surrounded by radish and cabbage plots, and the fungus was identified then [Marciniak and Macioszek, unpublished data]. Most lettuce plants were heavily infected and damaged at that time because the disease spread and decayed the whole plants. The contamination of the soil was caused by the spillage and plowing of the soil containing *S. sclerotiorum* derived from the tunnel lettuce cultivation carried out on the farm in early spring 2015. In 2016, lettuce was cultivated in the infected plots during the whole growing season without any additional or artificial inoculation of plants with the fungus.

The fungus was re-isolated from the infected lettuce heads of cultivar *Diamentinas*, harvested in early autumn 2015. The lettuce showed typical features for the late *S. sclerotinia* infection, with lower leaves wilted and covered with white mycelium and sclerotia and brownish rot tissues of the stem, with the blight separating the lettuce head off the stem. The samples of fungal mycelium were grown on PDA plates (Difco, Leeuwarden, The Netherlands) under laboratory conditions: temperature of 20 °C, approximately 65% humidity, and photoperiod 16 h day/8 h night under a fluorescent light 120 µmol m^−2^ s^−1^ for 14–21 days. After this time, a microscopic estimation of the fungal morphology was performed to evaluate whether the obtained fungus culture met Koch’s postulates. After 21 days, the whole PDA plates were covered with fungal culture comprising white mycelium (the same as the collected one) and many black hard sclerotia of 2–3 mm diameter, characteristic of *S. sclerotiorum*. Subsequently, Crispy lettuce heads of cultivar *Diamentinas* were infected with samples of the re-grown fungus under laboratory conditions. The same infection symptoms were observed as in the case of infected *Diamentinas* plants harvested for re-isolation of the fungus.

### 2.4. Evaluation of Lettuce Infection

The disease symptoms on plants in the infected and control plots were observed each week of cultivation. A plant was considered dead when almost all its leaves were wilted and brownish (Table 1). Individual plant survival was estimated every week of cultivation in each crop, and the percentage of surviving still-growing plants was calculated for each cultivar. In the case of disease progression, estimation of infection progression in lettuce cultivars at the 7th week was performed based on a 6-point scale (0–5) prepared for each lettuce cultivar grown in the early autumn crop (Table 1).

### 2.5. Analyses of Phenolics Contents

#### 2.5.1. Sample Collection and Preparation of Plant Extracts

Samples were harvested from the control and infected plants of each cultivar. However, two samples per leaf were harvested from the leaf blade and leaf base. In each crop, 12–50 samples from each treatment (control or infected) per each cultivar were collected.

About 100 mg samples were collected from each leaf and kept frozen at −80 °C until used. The samples were extracted in 1 mL of 80% methanol (HPLC grade, POCH, Gliwice, Poland) and centrifuged for 15 min at 14,000 rpm and temp. 4 °C. The supernatants were placed in new tubes and kept on ice. A total of 1 mL of methanol was added to the remaining pellets and vortexed. The samples were then centrifuged again. Thereafter, supernatants were added to the previously prepared ones and gently mixed. The obtained plant extracts were used to determine the total phenolic compounds (TPCs), flavonoid, and anthocyanin contents using a Power Wave plate spectrophotometer (BioTek Instruments, Winooski, VT, USA).

#### 2.5.2. Total Phenolic Compounds (TPCs)

Total phenolic compounds were determined using the Folin-Ciocalteau method, as described by Macioszek et al. [35]. Briefly, 100 μL of the plant extract was added to 3.85 mL of distilled water, and then 100 μL of the Folin-Ciocalteau reagent (Sigma-Aldrich, Poznan, Poland) was added. A blank (with 80% methanol instead of plant extract) was prepared in parallel. After 3 min of incubation, 1 mL of 10% sodium carbonate was added to each sample, thoroughly mixed, and incubated in the dark at room temperature for 30 min. Next, the samples were analyzed spectrophotometrically at a wavelength of 725 nm. The total phenolic compounds content was calculated using a gallic acid (Sigma-Aldrich, Poznan, Poland) calibration curve and expressed as mg g^−1^ FW.

#### 2.5.3. Flavonoids

The content of flavonoids in the plant extracts was determined as described by Macioszek et al. [35]. Briefly, 600 µL of plant extract, 1800 µL of 80% methanol, 120 µL of 10% aluminum chloride hexahydrate, and 120 µL of 1 M sodium acetate were mixed. After 30 min of incubation in the dark, the samples were transferred to a 96-well plate and analyzed spectrophotometrically at a wavelength of 415 nm. The content of flavonoids was calculated based on the absorbance of the standard quercetin solution (Sigma-Aldrich, Poznan, Poland) and expressed as mg g^−1^ FW.

#### 2.5.4. Anthocyanins

The content of anthocyanins was determined using an adapted method described by Lee et al. [36]. Briefly, 200 µL of plant extract was added to 800 µL of 0.025 M potassium chloride, pH 1, and mixed thoroughly. Another 200 µL of plant extract was added to 800 µL of 0.4 M sodium acetate, pH 4.5, and mixed thoroughly. The samples were incubated at room temperature for 30 min and analyzed spectrophotometrically at 520 and 700 nm wavelengths. The content of anthocyanins was calculated based on a cyanidin-3-glucoside equivalent and expressed in µg g^−1^ FW [36].

### 2.6. Statistical Analysis

All the statistical analyses, including analysis of variance (ANOVA) and a post hoc Duncan’s test (*p* < 0.05), were performed using Microsoft Office Excel 2021 (Microsoft Corporation, Redmond, WA, USA) and STATISTICA 13.3 (Tibco Software Inc., StatSoft, Krakow, Poland).

The charts were prepared using Microsoft Office Excel 2021. All the figures were composed using Adobe Photoshop CS3 version 10.0 (Adobe System Incorporated, San Jose, CA, USA).

## 3. Results

### 3.1. Changes in the Air and Soil Parameters

**Figure 2:**
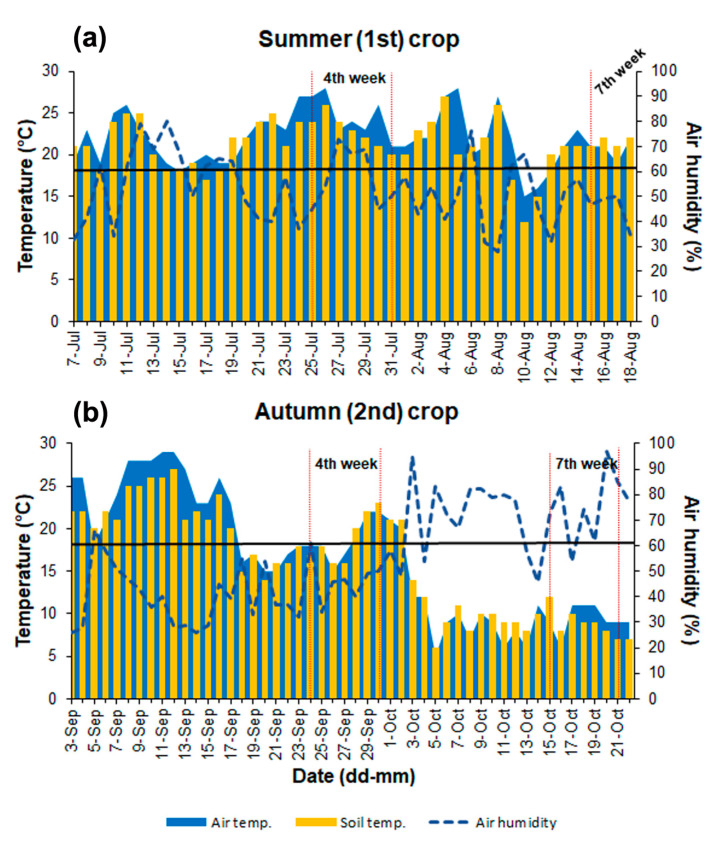
Changes in the soil and air temperature, and relative air humidity during vegetation seasons of lettuce in 2016. (**a**) The summer (first) crop; (**b**) early autumn (second) crop. The horizontal black line on each chart indicates a temperature of 18 °C, which is an optimal temperature for lettuce cultivation under field conditions in Poland. Detailed data are included in Supplementary Materials (Tables S1 and S2).

During two vegetation seasons in 2016, air temperature and humidity, as well as soil temperature and pH, were measured daily. In the summer crop (the first crop, 7 July–18 August), the range of air day temperatures was between 15 and 28 °C (Figure 2a). The temperature below the optimum (18 °C) for growing lettuce in the field was observed only for two days during the entire summer. The air temperature in the fourth week (when the first significant decrease in the survival of lettuces was observed) was high between 21 and 28 °C, but it only ranged between 19 and 22 °C at the end of the summer crop (Figure 2a, Table S1).

In the summer, the soil temperature for 21 days was lower, about 1–2 °C, than the air temperature, and it was higher than the air temperature only for 13 days. Air and soil temperatures were equal for the remaining seven days of the summer crop. The average air temperature in the summer was 22.0 °C, and the average soil temperature was 21.3 °C. Humidity was highly alterable during this period and ranged between 28 and 79%, with an average humidity of 52.9% (Figure 2a, Table S1).

In the autumn crop (the second crop, 3 September–22 October), the air temperature ranged between 5 and 29 °C (Figure 2b, Table S2). The lowest soil temperature was 6 °C, and the highest was 27 °C. Air and soil temperatures were lower in October (from the fifth week of this crop) than in September. The average air temperature in September was 21.7 °C and only 10.0 °C in October. The autumnal humidity ranged from 26 to 97%, with a significantly higher average value of 72% in October compared to only 41.6% in September. However, the average humidity of 55% in the autumn was only about 3% higher than in the summer crop (Figure 2b, Table S2). The temperature and humidity values remained moderately equal in the summer crop compared to variable ones in the autumn crop (Figure 2).

**Figure 3:**
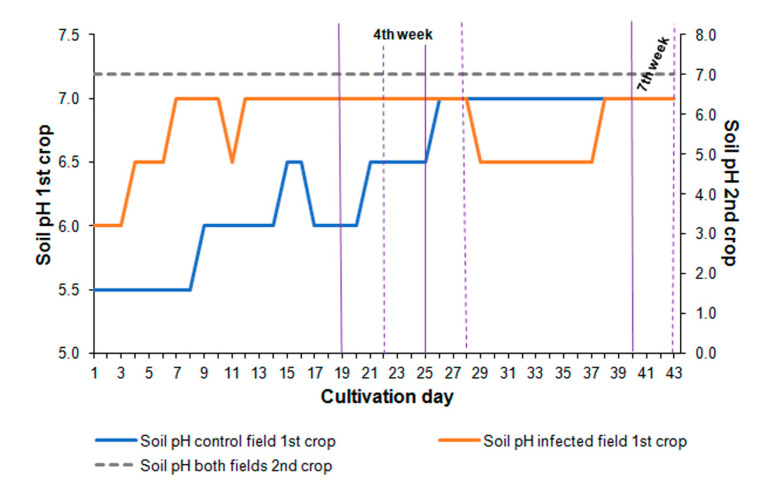
Average pH of the soil in the control and infected fields based on measurements taken daily during each vegetation season. Vertical solid violet lines indicate the fourth and seventh weeks during the summer crop, and vertical dashed violet lines indicate the corresponding weeks during the autumn crop. Detailed data are included in Supplementary Materials (Tables S1 and S2).

In the first summer crop, the pH of the soil in the control field for the first three weeks of cultivation was lower (pH 5.5–6.5) than that of the infected soil (pH 6.0–7.0). Three weeks after cultivation, the pH values equalized to a similar level in both fields. The average pH value of soil in the control field was 6.4 and 6.8 in the infected one during the summer crop. The soil pH remained constant (pH 7) during cultivation in the autumn crop, both in the case of control and infected fields (Figure 3).

Analysis of variance (one-way ANOVA) revealed that the soil and air temperatures, as well as soil pH, differed significantly between crops (*p* < 0.001). However, the humidity was not significantly variable between crops.

### 3.2. Re-Isolation of the Pathogen

**Figure 4:**
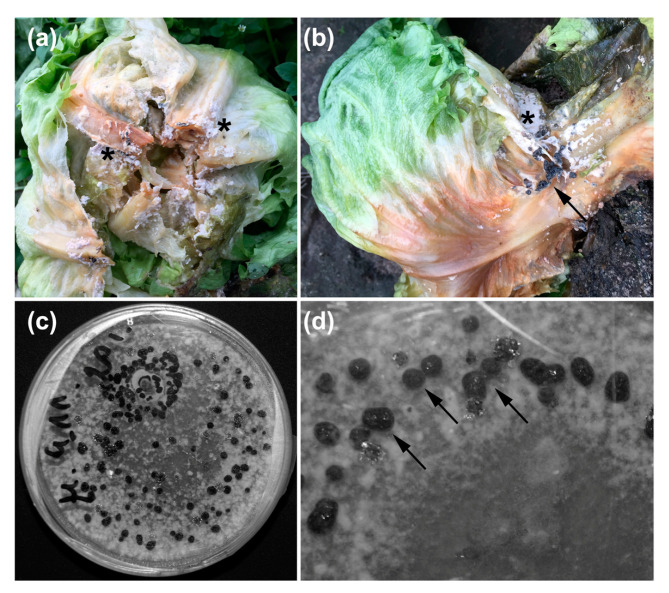
*Sclerotinia sclerotiorum* growth. (**a**,**b**) The fungus on heads of iceberg lettuce *Diamentinas* grown in the field in October 2015; (**c**,**d**) the re-isolated pathogen growing in vitro. Arrows indicate sclerotia and asterisks indicate white fluffy mycelium.

The pathogenic fungus was re-isolated from the infected lettuces (Figure 4a,b) and harvested in early autumn 2015. After 14–21 days of growth on the PDA plates under laboratory conditions, the fungus developed a fluffy white mycelium, accompanied by numerous black, hard sclerotia of 2–3 mm diameter (Figure 4c,d). The morphology of mycelium and sclerotia on plates was similar to that observed in infected lettuce heads, and based on this, the fungus was recognized as *S. sclerotiorum* [25]. Infection of healthy lettuce heads (cultivar *Diamentinas*) with the re-isolated fungus caused the same symptoms as in the case of infected lettuce from the autumn crop in 2015. Mature plant tissues were covered with white mycelium and sclerotia, and wilting leaves and stem rot were visible after 20 days of infection.

### 3.3. Evaluation of Disease Progression and Lettuce Survival

**Figure 5:**
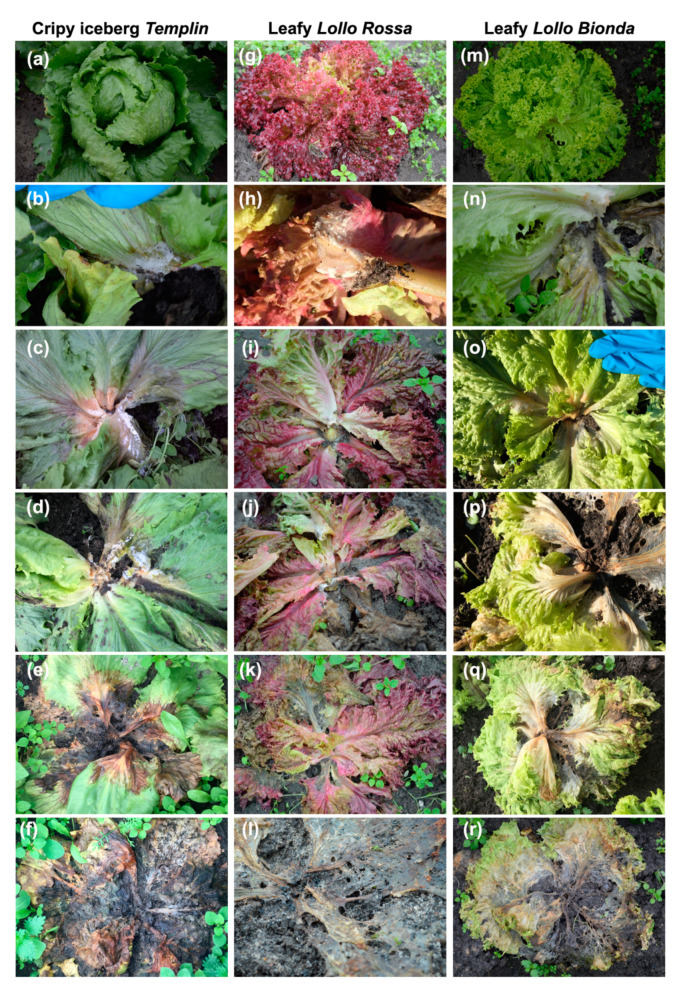
Progression of the disease symptoms in lettuce cultivars observed during the autumn crop. (**a**–**f**) Crispy iceberg lettuce *Templin*; (**g**–**l**) Leafy lettuce *Lollo Rossa*; (**m**–**r**) Leafy lettuce *Lollo Bionda*. Detailed description in the text.

In the control field, Crispy iceberg *Templin* plants formed compact heads on short stems, whereas both Leafy cultivars *Lollo Rossa* and *Lollo Bionda* grew, forming dense rosettes on short stems (Figure 5a,g,m). The infection of lettuce plants with *S. sclerotiorum* was observed exclusively in the infected field during both crops; there were no *S. sclerotiorum*-infected lettuces in the control field. The loss of plants in the control field was related to the abnormal, weak development of individual seedlings and the incidents of lettuce heads (*Templin*) being eaten by hares (Table 2 and Table 3).

The infection process in all lettuce cultivars began with the appearance of white mycelium on stems, leaf petioles, and bases close to the soil (stage 1, Table 1; Figure 5b,h,n). In the next second stage of disease development, wilting and browning of a few leaves and stems with watery spots were observed (Figure 5c,i,o). In the case of iceberg *Templin*, dark spots on leaf blades developed, and the spreading of mycelium from the stem to the leaves was visible (Figure 5c). The third stage characterized (Table 1) the first dark brown, decaying, death leaves that appeared in the plants of Crispy *Templin* and Leafy red *Lollo Rosa* (Figure 5d,j). Brown leaf petioles and discoloration of leaf blades could be observed in Leafy green *Lollo Bionda* plants at this stage of disease (Figure 5p). As the disease progressed, the disintegration of heads and rosettes could be found, and most of the leaves in *Templin* and *Lollo Rossa* plants were black and decoyed (Figure 5e,k). However, still-green leaves could be observed in *Lollo Bionda* plants (Figure 5q). The final fifth stage of the disease was characterized by discoloration and almost total decomposition of the infected plants of all three cultivars (Figure 5f,l,r). It has to be emphasized that such a sequence of disease symptoms similar to the ones described above appeared in all three cultivars during both crops.

In both vegetation seasons, the survival of individual plants of three planted cultivars was determined each week of cultivation in the control and infected fields. In the summer crop, the number of growing lettuce plants ranged between 80 and 100 per cultivar in the control field (Table 2). The lowest number of Crispy iceberg *Templin* lettuces was observed in the control field every week due to several lettuce heads eaten by hares. In the case of Leafy cultivars grown in the control field, at most, eight lettuce plants per cultivar were lost at the end of the summer crop. Overall, the cultivars grown in the control field showed significantly higher survival than lettuces planted in the one infected with *S. sclerotiorum* (Table 2). A gradual, significant decrease in survival of Crispy iceberg *Templin* was observed in the infected field every week. Beginning from the fourth week, when 80% of plants were still growing, the survival of this cultivar decreased to only 10% at the end of the summer crop (Table 2). The survival of Leafy green *Lollo Bionda* decreased only slightly up to the fourth week in both control and infected fields. However, there was a decline in the number of *Lollo Bionda* plants during the fifth week, with 79% of the plants surviving in the infected field compared to 92% still growing healthy plants in the control field. The downward trend in the survival of the *Lollo Bionda* cultivar continued until the seventh week of cultivation in the infected field, as only 70% of the lettuces ultimately survived. The leafy red lettuce *Lollo Rossa* showed the highest survival among lettuces planted in the infected field. Until the last week of cultivation, 83% of the plants of this cultivar still grew (Table 2).

In the autumn crop, 97% of Leafy red and green lettuce plants were still growing in the control field at the seventh final week of cultivation (Table 3). In the case of Crispy iceberg *Templin*, 92% of the healthy plants were noticed in the control field. A gradual decrease in the number of surviving plants in the infected field was observed for all the cultivars every week. The highest survival of 60% was observed for the Leafy green lettuce *Lollo Bionda*, and the lowest of 30% was observed for Crispy *Templin* at the end of the autumn crop. In the case of red lettuce *Lollo Rossa*, 51% of the plants survived the potential *S. sclerotiorum* infection in this crop (Table 3).

Analysis of the main effects of ANOVA revealed that the survival of all cultivars was highly dependent on both the crop and treatment (*p* < 0.05; Table S3). Furthermore, the survival of all lettuce cultivars in the infected field was correlated significantly to the soil and air temperatures and humidity (*p* < 0.05). However, only the survival of the *Lollo Rossa* was correlated to the soil pH in the infected field (*p* < 0.05) (Table S4). Interestingly, the survival of *Lollo Rossa* and *Lollo Bionda* was correlated to air and soil temperatures in the control field, and only *Lollo Rossa* survival was correlated significantly with humidity and soil pH (Table S5).

### 3.4. Analysis of Phenolics Content

In three lettuce cultivars, the contents of phenolic compounds, i.e., total phenolic compounds, flavonoids, and anthocyanins, were analyzed in control and infected samples. The infected samples were obtained from plants grown in the field with *S. sclerotiorum*-contaminated soil without additional plant inoculation. The control samples were collected from plants grown in the control field at corresponding sites as infected plants. The samples were obtained from the leaf base and leaf blade during the seventh week of cultivation in both crops.

#### 3.4.1. Total Content of Phenolic Compounds (TPCs)

**Figure 6:**
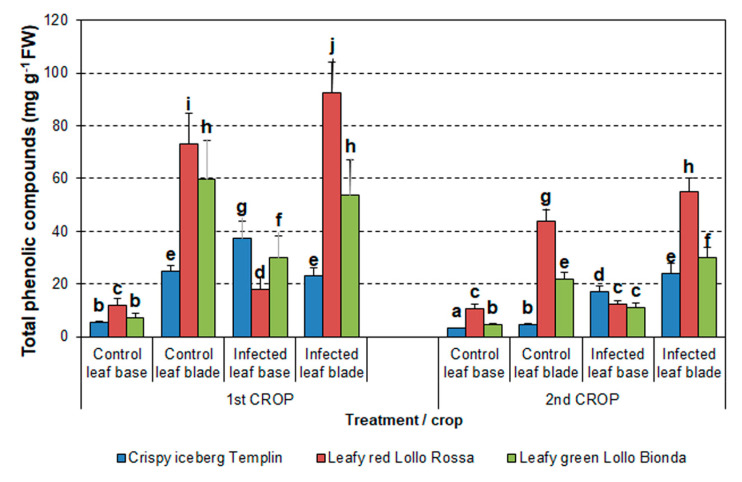
Total phenolic compounds content in lettuce cultivars in two vegetation seasons in 2016. The means ± SE were obtained from independent samples harvested from individual plants per treatment (n = 9–25). Different letters indicate a significant difference between the means according to Duncan’s test (*p* < 0.05).

Analysis of the samples of three lettuce cultivars during the first summer and second autumn crops revealed that the TPC content in the leaf base and leaf blade significantly differed (Figure 6).

In the first summer crop, the content of TPCs in infected plants was the lowest in both green lettuce cultivars *Templin* and *Lollo Bionda* compared to the control plants. The levels of TPCs in control and infected leaf blade samples from both cultivars did not differ, although the content of TPCs was higher in *Lollo Bionda* than in *Templin* (53.9–59.9 and 22.9–24.9 mg g^−1^ FW, respectively). A significant increase in TPC content was observed in infected samples of the red *Lollo Rossa* cultivar in the leaf base and blade compared to the corresponding control samples (Figure 6).

In the second autumn crop, the levels of TPCs significantly increased in the infected green lettuce cultivars *Templin* and *Lollo Bionda* in both the leaf base and leaf blade compared to the control plants. The highest levels of TPCs were observed in control and infected plants of the *Lollo Rossa* cultivar, compared to other cultivars. However, the highest levels of TPCs were observed in infected samples from the leaf blade of *Lollo Rossa* (55.05 ± 5.05 mg g^−1^ FW) in this crop. The content of TPCs in the leaf base of *Lollo Rossa* was at a similar level in the control and infected samples (Figure 6).

The TPC content was lower in all investigated lettuce cultivars from the second autumn crop than in plants from the first summer crop (F = 75.87; *p* < 0.001). Moreover, factorial analysis of variance revealed that TPC content was also significantly influenced by lettuce cultivar (F = 47.22, *p* < 0.001) and plant treatment (control/infected and leaf base/leaf blade, F = 85.29, *p* < 0.001).

#### 3.4.2. Flavonoid Content

**Figure 7:**
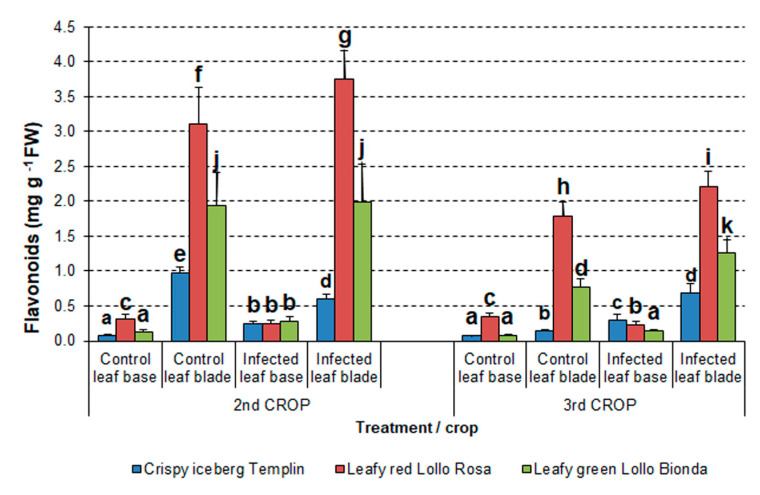
Changes in flavonoid content in lettuce cultivars in two vegetation seasons in 2016. The means ± SE were obtained from independent samples harvested from individual plants per treatment (n = 9–25). Different letters indicate a significant difference between the means according to Duncan’s test (*p* < 0.05).

As for TPC content, the flavonoid content in samples collected from the leaf blade was higher than in the leaf base. This trend was the case in both control and infected samples of all three cultivars during both crops, but a few exceptions were found (Figure 7). In the first summer crop, the flavonoid content of Crispy iceberg *Templin* was the lowest compared to other cultivars, despite treatment. Moreover, the levels of flavonoids in infected *Templin* plants were higher than those of control plants in samples obtained from the leaf base only. Surprisingly, the infected leaf blades of the *Templin* cultivar had significantly lower flavonoid content than the control samples. Flavonoid content increased significantly in the infected leaf base and leaf blade of Leafy green *Lollo Bionda* and red *Lollo Rossa* compared to the control samples. However, the levels of flavonoids did not differ in the infected leaf base and infected leaf blade of *Lollo Bionda* (Figure 7).

In the second autumn crop, the flavonoid content was higher in all infected samples of investigated cultivars obtained from the leaf blade compared to the control and leaf base samples. Only the *Lollo Rossa* samples obtained from the infected leaf base contained lower levels of flavonoids than the control. The flavonoid content in *Lollo Bionda* control and infected leaf blades was similar (Figure 7).

Similarly, as in the case of TPCs, factorial analysis of variance revealed that the flavonoid content was influenced significantly by crop (F = 47.9, *p* < 0.001), lettuce cultivar (F = 92.98, *p* < 0.001), and treatment (F = 147.6, *p* < 0.001). Flavonoid contents in the first summer crop were higher than in the second autumn crop for all cultivars.

#### 3.4.3. Anthocyanin Content

**Figure 8:**
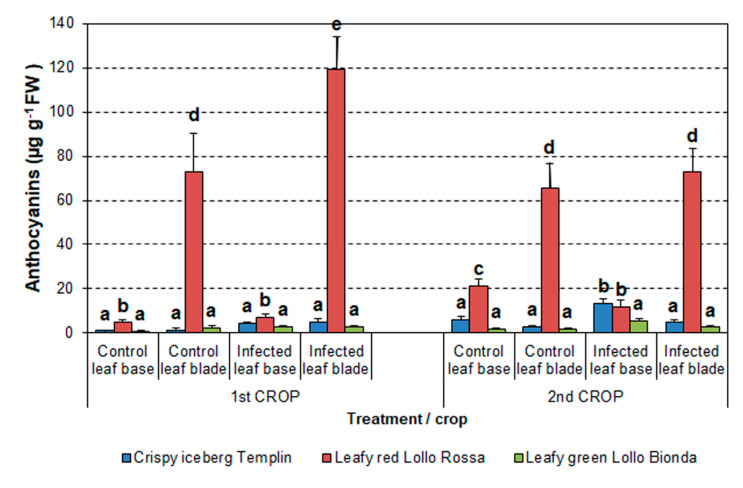
Differences in anthocyanin content in lettuce cultivars in two vegetation seasons in 2016. The means ± SE were obtained from independent samples harvested from individual plants per treatment (n = 3–24). Different letters indicate a significant difference between the means according to Duncan’s test (*p* < 0.05).

Analysis of anthocyanins in the first summer crop revealed that their contents did not change in the Crispy *Templin* and Leafy *Lollo Bionda* cultivars regardless of treatment (Figure 8). However, several times more anthocyanins were noticed in the leaf blades of the Leafy red *Lollo Rossa* cultivar than in both green cultivars. Furthermore, the anthocyanin levels in infected *Lollo Rossa* leaf blades significantly increased compared to the control. Similar trends in anthocyanin contents for all cultivars were observed in the second autumn crop (Figure 8).

Factorial analysis of variance indicated that the anthocyanin content was influenced significantly by lettuce cultivar (F = 97.33, *p* < 0.001) and treatment (F = 22.41, *p* < 0.001). Nevertheless, anthocyanin contents did not significantly differ between both crops.

## 4. Discussion

Lettuce is one of the most popular vegetables around the world. This leafy vegetable is investigated extensively in terms of improvement of its cultivation strategies to obtain high yields both in the field and indoors using various substrates or hydroponic culture [37,38,39]. Modifications to lettuce growth conditions to improve its qualities, resulting in a higher content of valuable nutrients and minerals, are also under study [40,41,42]. However, the extensive cultivation of crops as monocultures and the negative impact of global climate changes highly influence the quality and yield of crops, including lettuce. Moreover, these factors contribute to the increased activity of pathogens and pests, which threaten agriculture production, food security, and farmer incomes [42,43,44,45]. Simulation models predict that environmental conditions will impact especially incidents of soil-borne or air-borne fungus-induced diseases of crops and their severity due to the warming climate [46,47,48]. Forecasting models and research indicated temperature and humidity as factors influencing the development of *S. sclerotiorum* and the formation of ascospores in such crops as dry beans, canola, and lettuce. Higher average temperature (range of 15–25 °C) and humidity (above 90%) may intensify the development of the fungus on lettuce, extending the range of its occurrence [49,50,51]. In this study, we investigated the emergence of *S. sclerotiorum*-induced disease in three cultivars of lettuce grown on the market farm during two vegetation seasons, the summer and early autumn. Despite the stable temperature and humidity during the summer crop (over 18 °C, the optimal temperature for lettuce cultivation), their high variability in the autumn crop was observed. The average high air and soil temperatures in the first four weeks of the autumn crop (around 21 °C) were contrary to the much lower temperature in the last three weeks (around 10 °C). However, the severe disease symptoms (Figure 5) were observed first around the fourth week of each crop, exclusively on lettuces grown in the field with *S. sclerotiorum*-contaminated soil (Table 2 and Table 3).

The research on Polish isolates of *S. sclerotiorum* from canola has indicated that temperatures between 15 and 20 °C significantly accelerated the fungus development and sclerotia formation [52]. Sclerotia are produced most often during unfavorable environmental conditions or when nutrients are lacking. Several factors, such as temperature, humidity, and pH, can influence the formation and development of sclerotia [25]. Although it was not determined quantitatively, more sclerotia were observed at the end of the autumn crop, when the air and soil temperatures ranged between 6 and 14 °C (Figure 2). Moreover, *S. sclerotiorum* can grow and form sclerotia in a wide range of soil pH from 2.5 to 9 [26]. However, sclerotia formation in the culture at neutral or alkaline pH can be arrested [25]. Despite the fluctuating pH in the first summer crop and neutral pH in the autumn crop, the fungus developed extensively, causing severe disease symptoms and forming numerous sclerotia (Figure 3).

Furthermore, the development of lettuce drop depends on other factors, not only environmental conditions. It has been reported that host plants’ resistance to *Sclerotinia* spp. depends on their morphological and physiological attributions, such as lifting leaves off the soil or lignifying stems [53]. In our study, this phenomenon could also be noticed as Crispy iceberg *Templin* with watery leaves and a formed head had the lowest survival of 10–30% during both seasons, compared to Leafy lettuce cultivars forming rosettes such as *Lollo Bionda* and *Lollo Rossa* (Table 2 and Table 3). Moreover, various levels of commercial lettuce varieties/cultivars‘ resistance to *S. sclerotiorum* have been tested under field and growth room conditions [33], suggesting other physiological disease-restricting factors.

Individual lettuce cultivar features influencing the pathogen development and disease progression may be related to secondary metabolites such as phenolic compounds regarding their composition and content, similar to many other pathosystems [54,55]. Under normal growth conditions, lettuce leaf tissue contains a certain quantity of a wide range of phenolic compounds depending on the cultivar [56,57]. Environmental conditions affect the concentration of phenolic compounds in lettuce [12,13,15]. However, when exposed to stress conditions, the activity of the phenylpropanoid pathway intensifies, leading to the extensive biosynthesis and storage of various phenolic compounds such as flavonols and anthocyanins, protecting plants and reducing oxidative stress [15,58,59]. The lowest contents of total phenolic compounds and flavonoids were noticed in green lettuce cultivars Crispy iceberg *Templin* and Leafy *Lollo Bionda* in both vegetation seasons. In the case of red lettuce *Lollo Rosa*, the samples from infected plants had the highest content of phenolics (Figure 6 and Figure 7). Thus, activation of the phenylpropanoid pathway is closely related to enhanced resistance to *S. sclerotiorum* also in other plant species, such as a pea [60].

Purple-bluish anthocyanins are water-soluble pigment flavonoids, and they play a relevant role in lettuce defense against pathogenic fungi, showing strong antioxidant and antimicrobial properties [61,62]. Anthocyanin content is influenced by temperature and depends on a cultivar with higher content in red lettuce cultivars than green ones [62,63,64]. Accordingly, in our study, green lettuce cultivars *Templin* and *Lollo Bionda* contained low levels of anthocyanins compared to red lettuce *Lollo Rossa*. Moreover, we have shown first that anthocyanin content increased several times in *S. sclerotiorum*-infected lettuce *Lollo Rossa* compared to the control, while it remained at the same level or increased only slightly in infected green lettuce cultivars (Figure 8). Enhanced anthocyanin biosynthesis and accumulation have been reported in the wild-type Indian mustard infected with *S. sclerotiorum*, showing relatively small disease symptoms contrary to the spreading lesions in anthocyanin-devoid mutant plants [65]. One possible explanation of the antifungal effect of phenolics is the induction of cell death by phenolic acid (ferulic acid) described for the maize fungal pathogen *Cochliobolus heterostrophus* [66].

**Figure A2:**
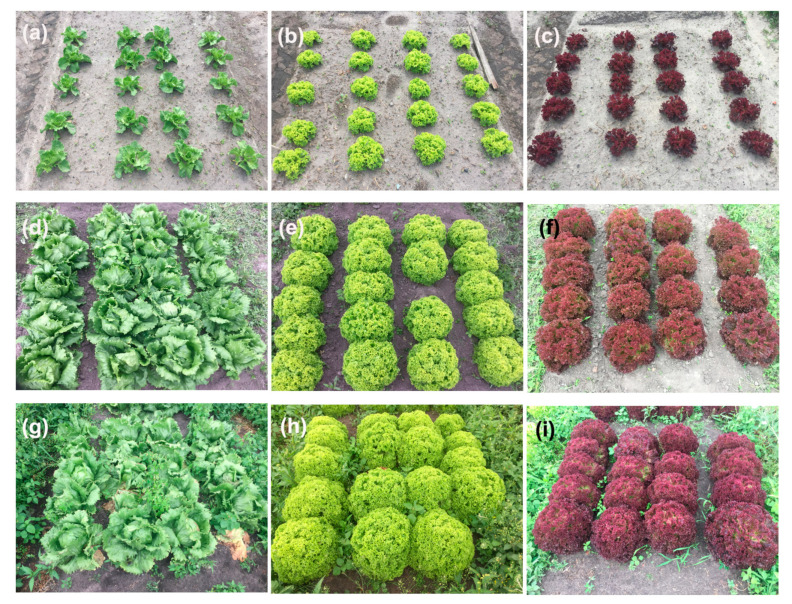
The lettuce crop was established on 30 April 2018, without any signs of *S. sclerotiorum* infection. Photos of lettuce cultivars in the infected field were taken on 15 May: (**a**) *Templin*; (**b**) *Lollo Bionda*; (**c**) *Lollo Rossa*; photos of lettuce cultivars in the infected field taken on 5 June: (**d**) *Templin*; (**e**) *Lollo Bionda*; (**f**) *Lollo Rossa*; photos of lettuce cultivars in the infected field taken on 18 June: (**g**) *Templin*; (**h**) *Lollo Bionda*; (**i**) *Lollo Rossa*.

Lettuce infection with *S. sclerotiorum* and severe symptoms of the lettuce drop incidents in many market farms limit agricultural production. The impact of this fungus on farm yield and economic performance prompted the development of research that would bring measurable benefits to farms enduring epidemics induced by *S. sclerotiorum*. In our case, however, the occurrence of a small-scale *S. sclerotiorum*-induced disease in lettuce was fought off and silenced with a crop rotation practice (Appendix A, Figure A2). However, the management of lettuce drop is mainly based on a few fungicides [67], and biocontrol methods are used, such as spraying plants with bacteria such as *Streptomycetes* or *Arthrobacter* [68,69]. Unfortunately, those methods are insufficient for combatting large-scale farm epidemics triggered by *S. sclerotiorum* [70]. Manipulating the content and/or composition of phenolic compounds using contemporary approaches may provide the foundation for modern management and defense strategy for successful lettuce cultivation [71].

## Figures and Tables

**Table 1:** The scale of disease progression in the tested lettuce cultivars by *S. sclerotiorum*.

Stage of Infection	Presence of the Fungus	Symptom on Plant
0	No fungus	No symptoms
1	Small white mycelium	Small necroses on the stem
2	White mycelium and black sclerotia on the stem	Wilted leaf base, necroses on leaves and stem
3	White mycelium and sclerotia on leaves and stems	Necroses on leaves and stems, marginal leaves wilted and brown
4	Mycelium spread and many black sclerotia	Wilted, rotten, and discolored leaves, rotten stem
5	Spreading fungus on the whole plant	Decoyed, dead plant

**Table 2:** Percentage of the survival of individual lettuce plants in the control and infected fields during the summer crop (July–August).

Cultivation Week	I	II	III	IV	V	VI	VII
Cultivar	Plants in control field (%)						
Crispy iceberg *Templin*	98	92 ^1^	90 ^1^	82 ^1^	80 ^1^	80 ^1^	80 ^1^
Leafy red *Lollo Rossa*	100	100	99	99	99	99	99
Leafy green *Lollo Bionda*	99	99	93	92	92	92	92
	Plants in infected field (%)						
Crispy iceberg *Templin*	100	97	95	80	70	21	10
Leafy red *Lollo Rossa*	97	96	90	89	89	84	83
Leafy green *Lollo Bionda*	100	94	93	93	79	70	70

^1^ Due to a few lettuce heads eaten by hares.

**Table 3:** Percentage of the survival of individual lettuce plants in the control and infected fields during the autumn crop (September–October).

Cultivation Week	I	II	III	IV	V	VI	VII
Cultivar	Plants in control field (%)						
Crispy iceberg *Templin*	100	100	100	98	96	96	92
Leafy red *Lollo Rossa*	100	100	100	100	99	99	97
Leafy green *Lollo Bionda*	100	100	100	99	99	99	97
	Plants in infected field (%)						
Crispy iceberg *Templin*	100	97	94	70	52	48	30
Leafy red *Lollo Rossa*	98	88	81	80	76	65	51
Leafy green *Lollo Bionda*	100	99	98	88	85	80	60

## Data Availability

Data are contained within the article.

## Supplementary Materials

The following supporting information can be downloaded at: https://www.mdpi.com/article/10.3390/pathogens12121416/s1. Table S1: Soil temperature and pH, and air temperature and humidity measured at 3 p.m. daily during the first crop from 7 July 2016 to 18 August 2016; Table S2: Soil temperature and pH, and air temperature and humidity measured at 3 p.m. daily during second crop from 3 September 2016 to 23 October 2016; Table S3. ANOVA of the lettuce survival during vegetation seasons; Table S4: Pearson’s correlations for the survival of lettuce cultivars with air and soil parameters in the infected field; Table S5: Pearson’s correlation coefficients for the survival of lettuce cultivars with air and soil parameters in the control field.

## Appendix A

Non-Evaluated Crops (Spring Crop 2016, Spring and Summer Crops 2017, and Spring Crop 2018).

In spring on 4 April 2016, the experimental crop was established with two lettuce cultivars, Crispy Iceberg *Diamentinas* and Leafy red *Lollo Rossa*. However, the crop was longer than the summer and autumn ones and lasted until 20 June (81 days) due to relatively low temperatures on days and nights. The evaluation of the disease progression and the collection of samples was carried out on the fourth and eleventh weeks of cultivation. This crop’s different establishment and longer cultivation period than the other 2016 crops made it impossible to correlate all three crops in 2016. Therefore, we have decided not to consider the spring crop in 2016 in our study (Figure A1).

In the spring and summer of 2017, Brussel sprouts plants instead of lettuce have been planted as a crop rotation practice in experimental plots. In early autumn 2017, the experimental plots were established, and the plant material comprised three lettuce cultivars: iceberg lettuce *Templin*, green lettuce *Lollo Bionda*, and red lettuce *Lollo Rossa*. However, it was impossible to collect enough samples from the fungus-infected and the control fields due to unfavorable weather conditions at that time (heavy rain).

Another lettuce crop was established on 30 April 2018, similar to the crops in 2016 and 2017. This crop lasted until 20 June 2018. However, there was no sign of any disease symptoms on any lettuce plants (Figure A2). Therefore, no samples were harvested, and no data were collected.
